# Integrated Sensing and Secure Communication with XL-MIMO

**DOI:** 10.3390/s24010295

**Published:** 2024-01-03

**Authors:** Ping Sun, Haibo Dai, Baoyun Wang

**Affiliations:** 1 School of Communication and Information Engineering, Nanjing University of Posts and Telecommunications, Nanjing 210023, China; 2018010214@njupt.edu.cn; 2 School of Internet of Things, Nanjing University of Posts and Telecommunications, Nanjing 210046, China; hbdai@njupt.edu.cn

**Keywords:** near field, integrated sensing and communication, beam focusing, vehicular ad hoc networks (VANETs)

## Abstract

This paper studies extremely large-scale multiple-input multiple-output (XL-MIMO)-empowered integrated sensing and secure communication systems, where both the radar targets and the communication user are located within the near-field region of the transmitter. The radar targets, being untrusted entities, have the potential to intercept the confidential messages intended for the communication user. In this context, we investigate the near-field beam-focusing design, aiming to maximize the achievable secrecy rate for the communication user while satisfying the transmit beampattern gain requirements for the radar targets. We address the corresponding globally optimal non-convex optimization problem by employing a semidefinite relaxation-based two-stage procedure. Additionally, we provide a sub-optimal solution to reduce complexity. Numerical results demonstrate that beam focusing enables the attainment of a positive secrecy rate, even when the radar targets and communication user align along the same angle direction.

## 1. Introduction

Integrated sensing and communication (ISAC) has emerged as a significant advancement in upcoming wireless systems [1,2]. These systems enable the dual use of hardware platforms and limited spectrum/power resources for both communication and sensing tasks [3]. One of the most promising areas of ISAC application is in *vehicular ad hoc networks* (VANETs), which are at the forefront of the evolution of connected and autonomous vehicles [4]. In VANETs, vehicles communicate with each other and possibly with infrastructure components, such as traffic signals or roadside units. These communications can be used for sharing traffic conditions, alerting nearby vehicles of emergency braking, or even for cooperative driving in the future [5]. With the increasing complexity and density of modern traffic systems, vehicles also need advanced sensing capabilities for safety and navigation purposes. By integrating ISAC into VANETs, vehicles can dynamically optimize resources, streamline hardware, and enhance safety through real-time communication of sensed data, ensuring efficient and adaptive network performance [6,7].

Currently, ISAC systems allow the reuse of information-bearing signals for radar sensing [2]. While this dual operation improves spectrum efficiency and reduces the cost of systems, it introduces potential risks of information leakage. This becomes particularly concerning when sensing targets, being untrusted entities, might intercept these confidential signals [8]. To address this issue, physical layer security techniques have been integrated into ISAC systems to ensure communication security [9,10,11]. In [9], the authors examine an ISAC scenario consisting of one communication user and one untrusted target, potentially an eavesdropper, and they design the transmit covariance matrices to maximize the achievable secrecy rate. In [10], the authors jointly optimize the transmit information and sensing beamforming, aiming to minimize the beampattern matching error for sensing while ensuring the minimum secrecy rate requirement. Meanwhile, in [11], optimization-based beamforming designs are presented, focusing on the security of information transmissions within ISAC systems.

It should be noted that all the aforementioned papers on secrecy ISAC address the far-field scenario, where both radar targets and communication users reside within the far-field region of the transmitter. Motivated by the great success of multiple-input multiple-output (MIMO) technology in 4G and 5G [12,13], in future 6G networks, extremely large-scale (XL)-MIMO is envisioned to be utilized. XL-MIMO, deploying hundreds, or even thousands, of antenna elements at base stations or reflecting elements at reconfigurable intelligent surfaces, can significantly improve performance, such as spectral efficiency [14]. The envisioned deployment of XL-MIMO in future 6G networks will likely lead to wireless operations in the radiating near-field (Fresnel) region, in contrast to conventional wireless systems, which typically operate in the far-field region [15]. Thus, there will be a shift in 6G: radar targets and communication users may predominantly operate within the near-field region of the transmitter [16]. While far-field propagation assumes a planar wavefront, the near-field region is characterized by a spherical wavefront. This unique near-field waveform propagation characteristic paves the way for the beam focusing technique, as detailed in [17,18], offering a notable boost in secrecy communication performance by mitigating information leakage. Nevertheless, this promising avenue has garnered limited attention in the current secrecy ISAC literature.

Motivated by the preceding discussion, in this paper, we explore the near-field beam-focusing design for secrecy ISAC systems. Here, one transmitter equipped with an extremely large-scale antenna array senses multiple radar targets and communicates with a single communication user concurrently. Both the radar targets and the communication users are situated within the near-field region of the transmitter. These radar targets, being untrusted, might eavesdrop on the confidential messages intended for the communication user. In this context, we investigate the near-field beam-focusing design to characterize the trade-off between radar sensing and secure communication. The main contributions of this paper are summarized below:

- To the best of the authors’ knowledge, this work represents the first study of the near-field secrecy ISAC scenario, with a focus on revealing the benefits of near-field operation for secure communication. To achieve this, we formulate a near-field secrecy beam-focusing problem to maximize the secrecy rate for the communication user while meeting the transmit beampattern gain requirement for each radar target. The considered problem is new and has not been previously studied in either the conventional far-field or near-field contexts.
- While the formulated problem is non-convex, we achieve a global optimum solution by employing a two-stage procedure based on semidefinite relaxation (SDR). Additionally, we propose a novel low-complexity sub-optimal beam-focusing design tailored for this new problem.
- Finally, we provide numerical results to validate the effectiveness of our proposed secrecy beam-focusing designs. Notably, our proposed beam-focusing designs enable a positive secrecy rate even when the radar targets and communication user align along the same angle direction, which is unattainable with far-field beam steering. Additionally, we illustrate the performance trade-off between radar sensing and secrecy communication in near-field ISAC systems.

The remainder of this paper is organized as follows: Section 2 introduces the system model and problem formulation for near-field secrecy ISAC systems. Section 3 presents the development of an optimal solution and a sub-optimal, lower-complexity alternative. In Section 4, we provide extensive simulation results to validate our designs and illustrate the trade-offs involved. Finally, Section 5 concludes the paper with a summary of our findings and potential future research directions.

Let boldface lower-case and upper-case letters denote vectors and matrices, respectively. Use ∥·∥ for the $\ell_{2}$ norm, ${\left(\cdot \right)}^{T}$ for the transpose, ${\left(\cdot \right)}^{H}$ for the Hermitian transpose, $\mathrm{Null}\left(\cdot \right)$ for the null space of a matrix, and C to represent complex numbers.

## 2. System Model and Problem Formulation

**Figure 1:**
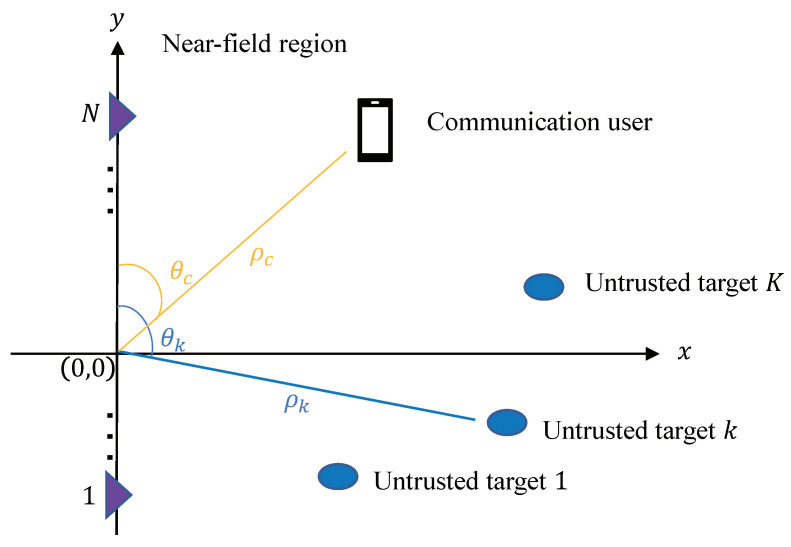
Illustration of near-field secrecy ISAC systems, consisting of one extremely large-scale antenna array that senses potential radar targets and serves communication users simultaneously.

Consider a near-field secrecy ISAC system, as illustrated in Figure 1. Here, one transmitter equipped with an extremely large-scale uniform linear array (ULA) of *N* antenna elements serves a single-antenna communication user and simultaneously senses *L* potential targets. Both the radar targets and the communication user are situated within the near-field region of the transmitter. These radar targets, being untrusted, might eavesdrop on the confidential messages intended for the communication user. With an inter-element spacing of *d*, the total antenna array aperture is $D=\left(N-1\right)d$. The boundary separating the near-field and far-field regions, known as either the Fraunhofer or Rayleigh distance, is defined as $d_{F}=\frac{2D^{2}}{\lambda }$ [19]. As the antenna count *N* increases, $d_{F}$ can extend over several hundred meters, encompassing numerous communication users and sensing targets. Given these considerations, research on near-field ISAC systems becomes increasingly crucial.

### 2.1. Near-Field Channel Model

The ULA of the transmitter is positioned along the *y*-axis. The Cartesian coordinate of the *n*th antenna element is represented as $p_{n}=\left(0,\left(n-\frac{N+1}{2}\right)d\right)$, with n=1,2,…,N. The communication user is located at $p_{c}=\left(\rho \sin\theta ,\rho \cos\theta \right)$, with ρ and θ denoting the distance and angle between the communication user and the array center, respectively. Let $d_{n}≜\sqrt{{\left(\rho \sin\theta \right)}^{2}+{\left(\rho \cos\theta -\left(n-\frac{N+1}{2}\right)d\right)}^{2}}$ denote the distance between the *n*th antenna element of the transmitter and the communication user. Denote *c* and $f_{c}$ as the light speed and the carrier, respectively. Then, the near-field channel between the transmitter and the communication user is given by

(1) $$h\left(\rho ,\theta \right)=ge^{-j2\pi f_{c}\frac{\rho }{c}}a\left(\rho ,\theta \right),$$

where $g≜\sqrt{{\left(\frac{c}{4\pi f_{c}d_{1}}\right)}^{2}}$, and a(ρ,θ) denotes the steering vector, given by

$$a\left(\rho ,\theta \right)\;=\;{\left[e^{-j2\pi f_{c}\frac{\left(d_{1}-\rho \right)}{c}},\cdots ,e^{-j2\pi f_{c}\frac{\left(d_{N}-\rho \right)}{c}}\right]}^{T}.$$

Let $\rho_{l}$ and $\theta_{l}$ denote the distance and angle between the *l*th untrusted radar target and the array center, respectively. The corresponding near-field channel between the transmitter and the *l*th untrusted radar target is given by

(2) $$h\left(\rho_{l},\theta_{l}\right)=g_{l}e^{-j2\pi f_{c}\frac{\rho_{l}}{c}}a\left(\rho_{l},\theta_{l}\right),\;1\leq l\leq L,$$

where $g_{l}≜\sqrt{{\left(\frac{c}{4\pi f_{c}d_{1}^{l}}\right)}^{2}}$, and the steering vector $a(\rho_{l},\theta_{l})$, given by

$$a\left(\rho_{l},\theta_{l}\right)\;=\;{\left[e^{-j2\pi f_{c}\frac{\left(d_{1}^{l}-\rho_{l}\right)}{c}},\cdots ,e^{-j2\pi f_{c}\frac{\left(d_{N}^{l}-\rho_{l}\right)}{c}}\right]}^{T}.$$

### 2.2. Signal Model

The transmitter uses beam focusing to transmit a combined signal for both communication and radar-sensing tasks. Represented by $x\in C^{N\times 1}$, the baseband signal at the transmitter is given by

(3) $$x=ws+t_{0},$$

where $w\in C^{N\times 1}$ is the beam-focusing vector for the communication user, with *s* being the information-bearing signal. On the other hand, $t_{0}$ is the dedicated radar signal with zero mean, and its covariance matrix is denoted as $R=E\left[t_{0}t_{0}^{H}\right]⪰0$.

According to (1) and (3), the received signal at the communication user can be written as $y_{c}=h^{H}\left(\rho ,\theta \right)\left(ws+t_{0}\right)+n_{c}$, where $n_{c}$ denotes the additive noise at the communication user, with zero mean and variance of $\delta_{c}^{2}$. Thus, the signal-to-interference-plus-noise ratio (SINR) at the communication user is expressed as

(4) $$\begin{array}{c} \Gamma_{c}\left(w,R\right)=\frac{{\left|h{\left(\rho ,\theta \right)}^{H}w\right|}^{2}}{h^{H}\left(\rho ,\theta \right)Rh\left(\rho ,\theta \right)+\sigma_{c}^{2}} \end{array}.$$

In the near-field ISAC scenarios, both communication and radar signals can contribute to radar-sensing tasks. Consequently, the transmit beampattern gain at the *l*th radar target is defined as [20]

(5) $$\begin{array}{cc} B_{l}\left(w,R\right) & =E\left[{\left|a^{H}\left(\rho_{l},\theta_{l}\right)\left(ws+t_{0}\right)\right|}^{2}\right]\\ \; & =a^{H}\left(\rho_{l},\theta_{l}\right)\left(ww^{H}+R\right)a\left(\rho_{l},\theta_{l}\right). \end{array}$$

The radar targets are untrusted nodes, which potentially intercept the confidential messages intended for the communication user. The SINR at the *l*th target is given by

(6) $$\begin{array}{c} \Gamma_{l}\left(w,R\right)=\frac{{\left|h{\left(\rho_{l},\theta_{l}\right)}^{H}w\right|}^{2}}{h^{H}\left(\rho_{l},\theta_{l}\right)Rh\left(\rho_{l},\theta_{l}\right)+\sigma_{l}^{2}},\;1\leq l\leq L. \end{array}$$

Based on (4) and (6), the achievable secrecy rate of the communication user can be expressed as

(7) $$\begin{array}{c} r\left(w,R\right)={\left[\log_{2}\left(1+\Gamma_{c}\left(w,R\right)\right)-\max_{1\leq l\leq L}\log_{2}\left(1+\Gamma_{l}\left(w,R\right)\right)\right]}^{+} \end{array},$$

where ${\left[x\right]}^{+}≜\max\left(0,x\right)$.

### 2.3. Problem Formulation

We are interested in characterizing the performance trade-off between radar sensing and secrecy communication. Specifically, we aim to maximize the secrecy rate of the communication user while satisfying the transmit beampattern gains for radar targets, by jointly optimizing the beam-focusing vector w and the radar covariance matrix R. Mathematically, the problem of interest is formulated as

(8) $$\begin{array}{cc} \max_{w,R}\; & r\left(w,R\right)\\ s.t.\;\; & B_{l}\left(w,R\right)\geq b_{l},\;1\leq l\leq L,\\ \; & {∥w∥}^{2}+\mathrm{Tr}\left(R\right)\leq P_{t},\;R⪰0, \end{array}$$

where $b_{l}$ denotes the transmit beampattern gain threshold for the *l*th radar target, and $P_{t}$ is the transmit power at the transmitter.

## 3. Proposed Solution

In this section, we first propose a two-stage optimization approach to solve the non-convex problem (8) globally optimally in Section 3.1. Then, in Section 3.2, we develop a low-complexity sub-optimal solution.

### 3.1. Optimal Solution

In this subsection, we present an SDR-based two-stage procedure to achieve the optimal solution for (8). This is accomplished by decomposing (8) into two sub-problems. First, by introducing a variable γ, we reformulate (8) as

(9) $$\begin{array}{cc} \max_{w,R}\; & \frac{{\left|h{\left(\rho ,\theta \right)}^{H}w\right|}^{2}}{h^{H}\left(\rho ,\theta \right)Rh\left(\rho ,\theta \right)+\sigma_{c}^{2}}\\ s.t.\;\; & \frac{{\left|h{\left(\rho_{l},\theta_{l}\right)}^{H}w\right|}^{2}}{h^{H}\left(\rho_{l},\theta_{l}\right)Rh\left(\rho ,\theta \right)+\sigma_{l}^{2}}\leq \gamma ,\;1\leq l\leq L,\\ \; & a^{H}\left(\rho_{l},\theta_{l}\right)\left(ww^{H}+R\right)a\left(\rho_{l},\theta_{l}\right)\geq b_{l},1\leq l\leq L,\\ \; & {∥w∥}^{2}+\mathrm{Tr}\left(R\right)\leq P_{t},\;R⪰0. \end{array}$$

Similar to [21], it can be proven that there always exists a γ for which problem (9) shares the same optimal solution as problem (8). To be specific, let $f\left(\gamma \right)$ denote the optimal value of (9) for a given γ>0. Consequently, the optimal value of (8) is equivalent to the following problem:

(10) $$\max_{\gamma >0}\;\log_{2}\left(\frac{1+f\left(\gamma \right)}{1+\gamma }\right)$$

Based on the above discussion, we conclude that problem (8) can be solved through a two-step process. Firstly, we solve (9) to obtain the optimal value $f\left(\gamma \right)$ for any given γ. Secondly, we solve (10) to find the optimal $\gamma^{*}$ by conducting a one-dimensional search over the parameter space. Consequently, we next focus on solving (9).

Problem (9) is still non-convex. Next, we apply the SDR technique to solve (9) globally optimally. To this end, by defining $W=ww^{H}$ and ignoring the non-convex rank-one constraint on W, we recast (9) as

(11) $$\begin{array}{cc} \max_{W,R}\; & \frac{\mathrm{Tr}\left[H(\rho ,\theta )W\right]}{\mathrm{Tr}\left[H(\rho ,\theta )R\right]+\sigma_{c}^{2}}\\ s.t.\;\; & \frac{\mathrm{Tr}\left[H(\rho_{l},\theta_{l})W\right]}{\mathrm{Tr}\left[H(\rho_{l},\theta_{l})R\right]+\sigma_{l}^{2}}\leq \gamma ,\;1\leq l\leq L,\\ \; & \mathrm{Tr}\left[A\left(\rho_{l},\theta_{l}\right)\left(W+R\right)\right]\geq b_{l},\;1\leq l\leq L,\\ \; & \mathrm{Tr}\left(W\right)+\mathrm{Tr}\left(R\right)\leq P_{t},\\ \; & \;W⪰0,\;R⪰0, \end{array}$$

where $H\left(\rho ,\theta \right)≜h\left(\rho ,\theta \right)h{\left(\rho ,\theta \right)}^{H}$, $H\left(\rho_{l},\theta_{l}\right)≜h\left(\rho_{l},\theta_{l}\right)h{\left(\rho_{l},\theta_{l}\right)}^{H}$, and $A(\rho_{l},\theta_{l})≜$$a\left(\rho_{l},\theta_{l}\right)a{\left(\rho_{l},\theta_{l}\right)}^{H}$.

Problem (11) is non-convex due to the non-concave nature of its objective function. Nevertheless, we can effectively address this by employing the Charnes–Cooper transformation [22], which allows us to equivalently reformulate (11) as

(12) $$\begin{array}{cc} \max_{\bar{W},\bar{R},\tau }\; & \mathrm{Tr}\left[H\left(\rho ,\theta \right)\bar{W}\right]\\ s.t.\;\; & \mathrm{Tr}\left[H\left(\rho ,\theta \right)\bar{R}\right]+\tau \sigma_{c}^{2}=1,\\ \; & \mathrm{Tr}\left[H\left(\rho_{l},\theta_{l}\right)\left(\bar{W}-\gamma \bar{R}\right)\right]\leq \tau \sigma_{l}^{2}\gamma ,1\leq l\leq L,\\ \; & \mathrm{Tr}\left[A\left(\rho_{l},\theta_{l}\right)\left(\bar{W}+\bar{R}\right)\right]\geq \tau b_{l},\;1\leq l\leq L,\\ \; & \mathrm{Tr}\left(\bar{W}\right)+\mathrm{Tr}\left(\bar{R}\right)\leq \tau P_{t},\\ \; & \;\bar{W}⪰0,\;\bar{R}⪰0,\tau >0. \end{array}$$

Problem (12) is convex, and thus, it can be solved directly using existing solvers such as CVX [23].

Let $\left\{{\bar{W}}^{*},{\bar{R}}^{*},\tau^{*}\right\}$ represent the optimal solution to (12). It can be readily demonstrated that $W^{*}=\frac{{\bar{W}}^{*}}{\tau^{*}}$ and $R^{*}=\frac{{\bar{R}}^{*}}{\tau^{*}}$ are the optimal solution to (11). If $\mathrm{Rank}\left(W^{*}\right)=1$, the relaxation of the rank-one constraint in (11) does not impact its optimality. We now present a theorem affirming that we can always construct an optimal solution to (12) with a rank-one matrix $\bar{W}$.

**Theorem** **1.** *Suppose that $\left\{{\bar{W}}^{*},{\bar{R}}^{*},\tau^{*}\right\}$ represents the optimal solution to problem (12) with $\mathrm{Rank}\left({\bar{W}}^{*}\right)>1$. Then, we can construct an alternative feasible solution to (12), denoted as $\left\{{\tilde{W}}^{*},{\tilde{R}}^{*},{\tilde{\tau }}^{*}\right\}$. This alternative solution not only attains an equivalent objective value as $\left\{{\bar{W}}^{*},{\bar{R}}^{*},\tau^{*}\right\}$, but also satisfies $\mathrm{Rank}\left({\tilde{W}}^{*}\right)=1$.*

**Proof of Theorem** **1.** The Lagrangian of problem (12) is given by

(13) $$L=\mathrm{Tr}\left(\left(A+Z\right)\bar{W}\right)+\mathrm{Tr}\left(B\bar{R}\right)+\rho \tau +\mu ,$$

where Z⪰0 is a matrix Lagrange multiplier associated with the constraint $\bar{W}⪰0$; A, B, and ρ are, respectively, defined as

(14) $$A\;=\;H\left(\rho ,\theta \right)\;-\;\sum_{l=1}^{L}\beta_{l}H\left(\rho_{l},\theta_{l}\right)\;+\;\sum_{l=1}^{L}\eta_{l}A\left(\rho_{l},\theta_{l}\right)\;-\;\nu I,$$

(15) $$B\;=\;-\mu H\left(\rho ,\theta \right)\;+\;\sum_{l=1}^{L}\beta_{l}\gamma H\left(\rho_{l},\theta_{l}\right)\;+\;\sum_{l=1}^{L}\eta_{l}A\left(\rho_{l},\theta_{l}\right)\;-\;\nu I,$$

(16) $$\rho =-\mu \;+\;\sum_{l=1}^{L}\beta_{l}\sigma_{l}^{2}\;-\;\sum_{l=1}^{L}\eta_{l}b_{l}\;+\;\nu P_{t},$$

where μ≥0, $\left\{\beta_{l}\geq 0\right\}$, $\left\{\eta_{l}\geq 0\right\}$, and ν≥0 represent the Lagrange multipliers corresponding to the first through fourth constraints in (12), respectively.

Given (13), the relevant Karush–Kuhn–Tucker (KKT) conditions for the proof are formulated as

(17a) $$\begin{array}{c} \frac{\partial L}{\partial \bar{W}}=A^{*}+Z^{*}=0, \end{array}$$

(17b) $$\begin{array}{c} Z^{*}{\bar{W}}^{*}=0, \end{array}$$

(17c) $$\begin{array}{c} \mu^{*},\beta_{l}^{*},\eta_{l}^{*},\nu^{*}\geq 0, \end{array}$$

where $Z^{*}$, $\mu^{*},\beta_{l}^{*},\eta_{l}^{*},\;\mathrm{and}\;\nu^{*}$ denote the optimal Lagrange multipliers for the dual problem of (12), and the resulting A and B are $A^{*}$ and $B^{*}$. Here, (17b) is obtained from the complementary slackness condition.

According to $Z^{*}⪰0$, (17a) and (17b), we have

(18a) $$\begin{array}{c} A^{*}⪯0 \end{array}$$

(18b) $$\begin{array}{c} A^{*}{\bar{W}}^{*}=0. \end{array}$$

Define $C^{*}\;=\;-\sum_{l=1}^{L}\beta_{l}^{*}H\left(\rho_{l},\theta_{l}\right)\;+\;\sum_{l=1}^{L}\eta_{l}^{*}A\left(\rho_{l},\theta_{l}\right)\;-\;\nu^{*}I$. Then, we have

(19) $$A^{*}=C^{*}+H\left(\rho ,\theta \right).$$

Based on (18b), the columns of ${\bar{W}}^{*}$ must lie in the null space of $A^{*}$ when ${\bar{W}}^{*}\neq 0$. Define $M≜\mathrm{Rank}\left(C^{*}\right)$. If M=N, we can conclude that $\mathrm{Rank}\left(A^{*}\right)\geq \mathrm{Rank}\left(C^{*}\right)-\mathrm{Rank}\left(H\right)=N-1$. In this case, we have $\mathrm{Rank}\left({\bar{W}}^{*}\right)=1$ when ${\bar{W}}^{*}\neq 0$.

We next discuss the case of M<N. In this case, let $X\in C^{N\times \left(N-M\right)}$ denote the orthonormal basis of the null space of $C^{*}$, i.e., $C^{*}X=0$. Let $x_{m},1\leq m\leq \left(N-M\right)$, denote the *m*th column of X. For each $x_{m}$, it follows

(20) $$x_{m}^{H}A^{*}x_{m}=x_{m}^{H}C^{*}x_{m}+x_{m}^{H}Hx_{m}=x_{m}^{H}Hx_{m}⪯0.$$

From (20), we conclude that $x_{m}^{H}Hx_{m}=0$. Thus, we have

(21) $$N-M+1\geq \mathrm{Rank}\left(\mathrm{Null}\left(A^{*}\right)\right)\geq N-M.$$

If $\mathrm{Rank}\left(\mathrm{Null}\left(A^{*}\right)\right)=N-M$, we have $\mathrm{Null}\left(A^{*}\right)=X$. This means ${\bar{W}}^{*}$ can be expressed as ${\bar{W}}^{*}=\sum_{m=1}^{M}a_{m}x_{m}x_{m}^{H}$. However, since $x_{m}^{H}Hx_{m}=0$, in this case, the achievable secrecy rate is equal to zero. Thus, we obtain $\mathrm{Rank}\left(\mathrm{Null}\left(A^{*}\right)\right)=N-M+1$. As a result, the optimal ${\bar{W}}^{*}$ can be expressed as

(22) $${\bar{W}}^{*}=\sum_{m=1}^{M}a_{m}x_{m}x_{m}^{H}+duu^{H},$$

where $a_{m}\geq 0,\forall m$ and d>0. u is an extra dimension of orthonormal basis, which lies in the null space of $A^{*}$ and is orthogonal to the span of X, i.e., $A^{*}u=0$ and Xu=0.

Based on (22), for the case of $\mathrm{Rank}\left({\bar{W}}^{*}\right)>1$, we can construct another feasible solution to problem (12), given by

(23) $${\tilde{W}}^{*}={\bar{W}}^{*}-\sum_{m=1}^{M}a_{m}x_{m}x_{m}^{H}=duu^{H},$$

(24) $${\tilde{R}}^{*}={\bar{R}}^{*}+\sum_{m=1}^{M}a_{m}x_{m}x_{m}^{H},$$

(25) $${\tilde{\tau }}^{*}=\tau^{*}.$$

It can be easily verified that $\left\{{\tilde{W}}^{*},{\tilde{R}}^{*},{\tilde{\tau }}^{*}\right\}$ is a feasible solution to problem (12), which can not only achieve the same objective value as $\left\{{\bar{W}}^{*},{\bar{R}}^{*},\tau^{*}\right\}$, but also satisfies $\mathrm{Rank}\left({\tilde{W}}^{*}\right)=1$. The proof of the optimality of $\left\{{\tilde{W}}^{*},{\tilde{R}}^{*},{\tilde{\tau }}^{*}\right\}$ in problem (12) is thus completed. □

Theorem 1 indicates that the optimal solution $w^{*}$ to (9) can be accurately extracted by the eigenvalue decomposition of $W^{*}$, with $W^{*}=\frac{{\tilde{W}}^{*}}{{\tilde{\tau }}^{*}}$. Consequently, $\left\{w^{*},{\tilde{R}}^{*}\right\}$ are the globally optimal solution to problem (9).

### 3.2. ZF-Based Sub-Optimal Solution

In this subsection, we propose a sub-optimal solution based on zero-forcing (ZF) for problem (8), which exhibits a significantly lower computational complexity compared to the optimal solution. This sub-optimal approach involves aligning the information beam w with the null space of the radar target’s channel to prevent any information leakage to potential eavesdroppers, as expressed by $h{\left(\rho_{l},\theta_{l}\right)}^{H}w=0,1\leq l\leq L$. Simultaneously, the radar signal is constrained to lie within the null space of the communication user, specified as $h^{H}\left(\rho ,\theta \right)Rh\left(\rho ,\theta \right)=0$, ensuring that it does not interfere with the communication user.

Define $H≜{\left[h\left(\rho_{1},\theta_{1}\right),\cdots ,h\left(\rho_{L},\theta_{L}\right)\right]}^{H}$. Denote its singular value decomposition as $H=U\Sigma V^{H}=U\Sigma {\left[V_{1},V_{2}\right]}^{H}$, where $U\in C^{L\times L}$ and $V\in C^{N\times N}$ are unitary matrices, Σ is a L×N diagonal matrix, $V_{1}\in C^{N\times L}$ and $V_{2}\in C^{N\times (N-L)}$ are the first *L* and the last N−L right singular vectors of H. In order to guarantee $h{\left(\rho_{l},\theta_{l}\right)}^{H}w=0,1\leq l\leq L$, the ZF-based w has the following form:

(26) $$w=\sqrt{p_{c}}V_{2}\tilde{w},$$

where $p_{c}$ denotes the transmit power of the information beam, and $\tilde{w}\in C^{(N-L)\times 1}$ is an arbitrary complex vector of unit norm. To maximize the secrecy rate, $\tilde{w}$ should match the equivalent channel $h{\left(\rho ,\theta \right)}^{H}V_{2}$, and thus $\tilde{w}=\frac{V_{2}^{H}h\left(\rho ,\theta \right)}{∥V_{2}^{H}h\left(\rho ,\theta \right)∥}$.

According to (26), the achievable secrecy rate becomes

(27) $$r_{\mathrm{ZF}}=\log_{2}\left(1+\frac{p_{c}{∥h{\left(\rho ,\theta \right)}^{H}V_{2}∥}^{2}}{\sigma_{c}^{2}}\right).$$

To maximize the secrecy rate in (27), we need to minimize the power consumption required to ensure the radar-sensing task. Consequently, we can obtain the optimal radar-sensing covariance matrix R by solving the following problem:

(28) $$\begin{array}{cc} \; & \min_{R}\;\mathrm{Tr}\left(R\right)\\ \; & \;s.t.\;\;\;\;a^{H}\left(\rho_{l},\theta_{l}\right)Ra\left(\rho_{l},\theta_{l}\right)\geq b_{l},\;1\leq l\leq L,\\ \; & \;\;\;\;\;\;\;\;\;\;h^{H}\left(\rho ,\theta \right)Rh\left(\rho ,\theta \right)=0. \end{array}$$

Problem (28) is convex and can be efficiently solved using CVX [23]. Let $R^{*}$ represent the optimal solution to (28). Consequently, the transmit power $p_{c}$ in (26) or (27) can be expressed as $p_{c}=P_{t}-\mathrm{Tr}\left(R^{*}\right)$.

## 4. Simulation Results and Discussion

In this section, we showcase numerical results to validate our secure beam-focusing design in the context of near-field ISAC systems. Our system setup involves a transmitter with a ULA of N=64 antennas, operating at 2.4 GHz. The Rayleigh distance for this setup is $d_{F}=248$ m. We position the communication user at (32,0) meters on the *x*-axis. All receivers are assumed to have an identical noise power of $\sigma_{c}^{2}=\sigma_{1}^{2}=\cdots =\sigma_{L}^{2}=-95$ dBm.

**Figure 2:**
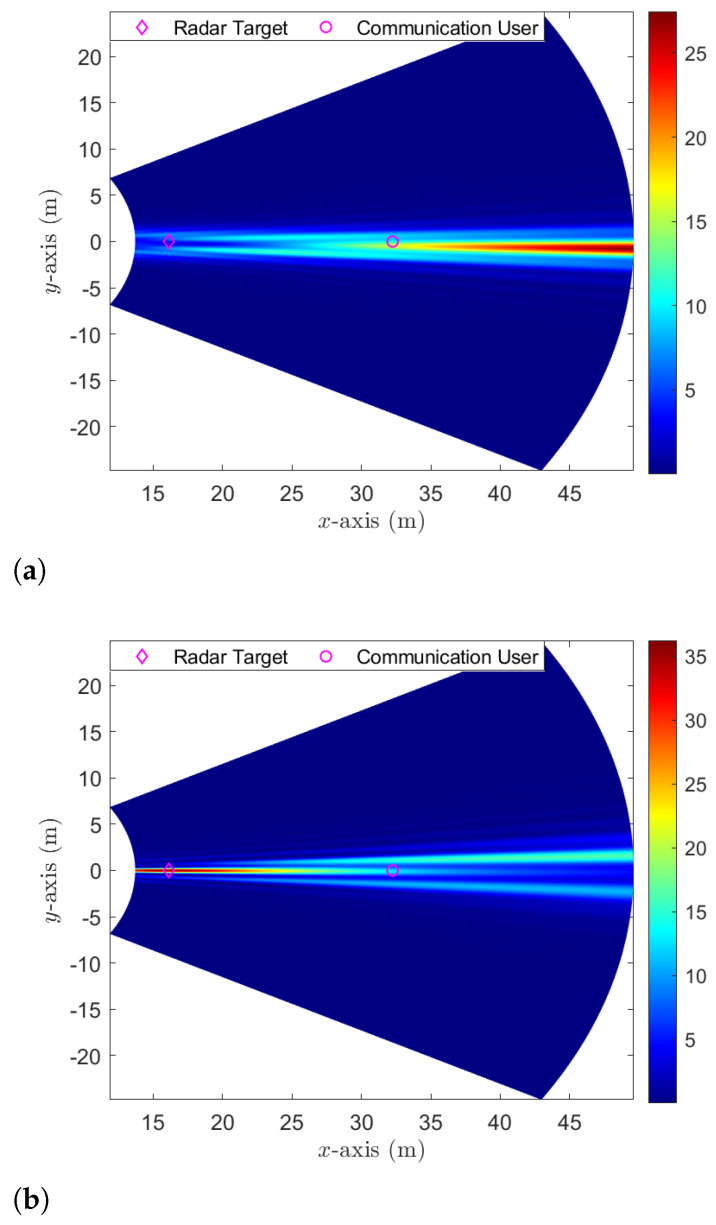
The normalized signal power of communication and radar signals. (**a**) Communication signal; (**b**) radar signal.

We first show the capabilities of beam focusing for ensuring secure communication. For our demonstration, we consider that a single radar target, which can potentially act as an eavesdropper, is positioned at (16,0) meters. This location coincides with the angular direction of the communication user. Figure 2a,b show the normalized signal power of the communication signal and radar signal at each point of the near-field xy-plane, respectively, given the transmit power $P_{t}=1$ dB and the transmit beampattern gain threshold $b_{l}=40,\forall l$. Both the communication signal beam w and radar signal covariance matrix R are obtained by solving problem (8) based on the given user locations and the predefined threshold values. From Figure 2a, we observe that most of the energy from the communication beam w is concentrated around the communication user. This results in minimal information leakage to the radar target. In contrast, Figure 2b shows that the energy from the radar covariance matrix R predominantly focuses on the radar target, ensuring limited interference to the communication user. Analyzing both Figure 2a,b, we conclude that our proposed secure beam focusing is adept at facilitating secure communication even when the radar targets and communication user occupy the same angular position—a feat not feasible with traditional far-field beam steering.

**Figure 3:**
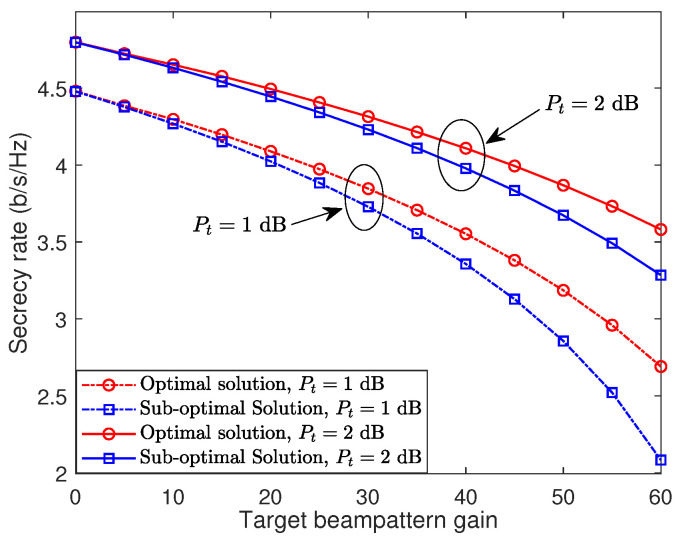
Trade-off between the secrecy rate and the transmit beampattern gain.

Figure 3 depicts the trade-off between the achievable secrecy rate and the transmit beampattern gain, as evaluated using both the optimal and sub-optimal solutions. The simulation settings are consistent with those used in Figure 2. It is observed that, as the target beampattern gain requirement rises, the achievable secrecy rate declines sharply. This decline occurs because with a fixed and limited total transmit power, dedicating more power to radar sensing reduces the power available for secure communication. This observation indicates that careful design of the secure beam-focusing scheme is crucial for balancing these two performances. Additionally, the optimal solution consistently surpasses the sub-optimal solution regarding the trade-off between secrecy rate and target beampattern gain. This superior performance stems from the more comprehensive and precise optimization techniques used in deriving the optimal solution, albeit at the cost of increased computational complexity. As anticipated, increasing the transmit budget enhances either the secrecy rate or the target beampattern gain.

**Figure 4:**
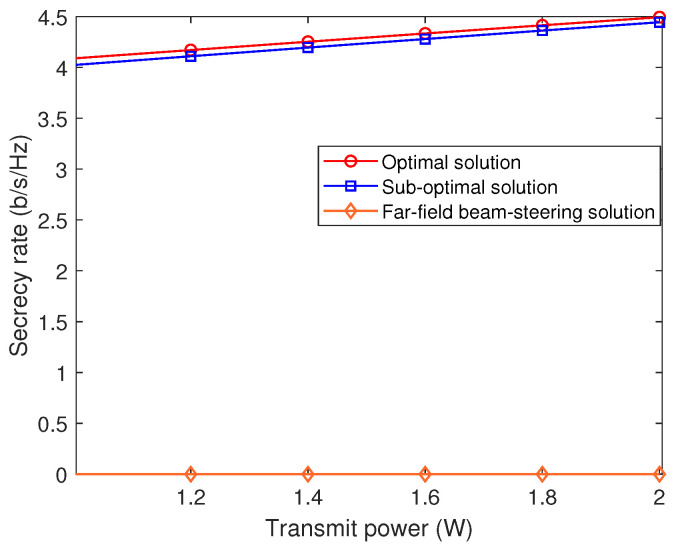
Secrecy rate versus the transmit power.

Figure 4 effectively illustrates the relationship between the achievable secrecy rate and transmit power, evaluated using both the optimal and sub-optimal near-field solutions, along with the far-field beam steering solution. The far-field beam steering solution is derived by substituting the precise near-field channel model with a standard far-field channel model, thereby neglecting the effects of the near-field. The simulation settings are consistent with those used in Figure 2. Figure 4 shows that the secrecy rates achievable by both the optimal and sub-optimal near-field solutions increase as the transmit power increases. In contrast, the achievable rate of the far-field beam steering remains consistently at zero. This is due to the untrusted radar target, potentially an eavesdropper, sharing an identical angular direction with the communication user, coupled with a relatively smaller distance from the transmitter. Consequently, this potential eavesdropper experiences superior channel conditions compared to the legitimate communication user. Under these circumstances, far-field beam steering fails to differentiate between the untrusted radar target and legitimate communication users, resulting in a zero secrecy rate. Conversely, near-field solutions yield positive secrecy rates, thanks to the beam-focusing capability in the near-field, which proficiently distinguishes users in both angular and distance dimensions.

**Figure 5:**
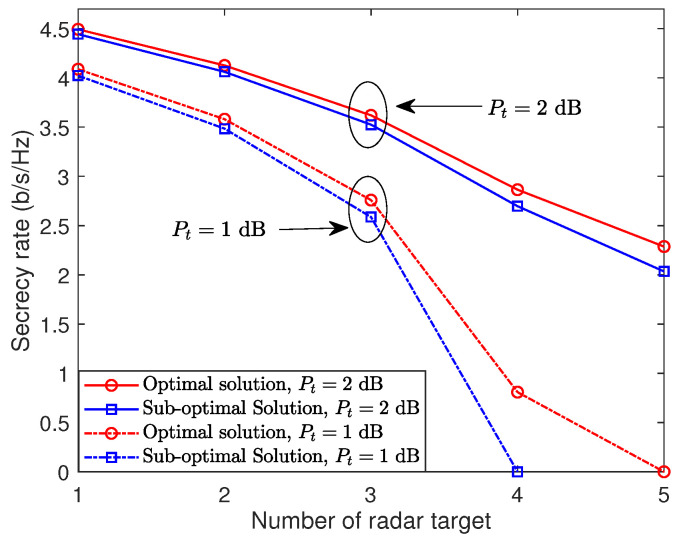
Secrecy rate versus the number of radar targets.

Finally, we study a general scenario with varying numbers of radar targets randomly positioned on the near-field xy-plane. Figure 5 shows a plot of the achievable secrecy rate against the number of radar targets *L*, with a consistent radar target threshold $b_{l}=20,\forall l$. We successively add radar targets to the near-field xy-plane. As seen in Figure 5, the achievable secrecy rate for both optimal and sub-optimal solutions consistently decreases as the number of radar targets grows. This is because, as the quantity of radar targets rises, so does the likelihood of intercepted communications, consequently leading to a reduced achievable secrecy rate. Additionally, a higher transmit power can improve the secrecy rate for a given radar target count.

## 5. Conclusions

This paper addresses beam-focusing design in near-field ISAC systems using XL-MIMO, focusing on maximizing the secrecy rate while meeting radar target requirements. It introduces both globally optimal and practical low-complexity sub-optimal solutions, catering to different computational capacities. Our numerical results demonstrate the efficacy of these solutions, notably achieving positive secrecy rates in challenging scenarios where the radar targets and communication user are positioned at identical angles. This study thus marks a significant advancement in secure communication within near-field ISAC environments, showcasing the potential of XL-MIMO in overcoming traditional security challenges.

One interesting extension of this paper is to use the Cramér–Rao bound (CRB) to evaluate the estimation accuracy. In such a case, the beam-focusing vectors and the radar covariance matrix should be jointly optimized to minimize the CRB. Furthermore, the beam-focusing design can also be extended to the robust beam-focusing case, where perfect channel state information is not necessary. Additionally, while our proposed algorithms have been effectively validated through simulations, we recognize the need for their verification through practical measurements in future research. This step is crucial for a comprehensive assessment of their real-world applicability and performance.

## Data Availability

Data is contained within the article.
